# Radical prostatectomy for high-risk prostate cancer | *Opinion: NO*

**DOI:** 10.1590/S1677-5538.IBJU.2019.03.03

**Published:** 2019-07-27

**Authors:** Saum Ghodoussipour, Giovanni Enrico Cacciamani, Andre Luis de Castro Abreu

**Affiliations:** 1 USC Institute of Urology and Catherine & Joseph Aresty Department of Urology, University of Southern California, Los Angeles, CA, USA

**Keywords:** Prostatic Neoplasms, Prostatectomy, Radiotherapy

## BACKGROUND

Prostate cancer (PC) is the most common solid malignancy in men. In 2019, there are expected to be 174,000 new diagnoses in the United States with 31,000 patients ultimately succumbing to their disease (1). Those with more aggressive disease are at a greater risk of local treatment failure and death (2), thus emphasis on the appropriate management for the subset of patients with high risk PC (HRPC) is paramount.

Current guideline recommendations for patients with HRPC include radical prostatectomy (RP), external beam radiotherapy (EBRT) with androgen deprivation therapy (ADT) or EBRT plus brachytherapy (BT) and ADT (3–6). Each guideline panel separately defines the criteria that place patients at higher risk of adverse outcomes. These criteria include a combination of preoperative prostate specific antigen (PSA), biopsy Gleason score and clinical stage (Table-1). Herein, it is our goal to highlight the differences in outcomes amongst these treatment options, the heterogeneity that exists within the HRPC category and to show where the evidence for treatment of HRPC with surgery is lacking.

**Table 1:** High Risk Prostate Cancer definition criteria according to different Guidelines panels.

	High-Risk PC		Very High-Risk PC
	**Localized**	**Non Localized**	Not defined
EAU/ESTRO/ESUR/SIOG Guidelines	PSA > 20 ng/ml *or* GS > 7 (ISUP Grade 4/5) *or* cT2c	any PSA *any* GS (*any* GS grade) cT3-4 or cN+	
	**Localized**		Not defined
AUA/ASTRO/SUO Guidelines	PSA > 20 ng/ml *or* GS > 7 (ISUP Grade 4/5) *or* ≥ cT3		
NCCN Guidelines	**Localized**		Localized
	PSA > 20 ng/ml *or* GS > 7 (ISUP Grade 4/5) *or* cT3a		cT3b-cT4 or Primary Gleason pattern 5 or > 4 cores with GS > 7

**EAU** = European Association of Urology; **ESTRO** = European Society for Radiotherapy & Oncology; **SIOG** = International Society of Geriatric Oncology; **AUA** = American Urological Association; **ASTRO** = American Society for Radiation Oncology; **SUO** = Society of Urologic Oncology; **NCCN** = National Comprehensive Cancer Network. **PSA** = Prostate Specific Antigen; **GS** = Gleason score; **ISUP** = International Society of Urological Pathologists.

### A role for definitive treatment

In 2012, results from the PIVOT trial exposed the heterogeneity of PC. Men with low risk disease appeared to have long-term disease specific survival whether or not they underwent surgery or watchful waiting. However, those with higher risk disease were at greater risk of developing bone metastases or death without surgery (7). The SPCG-4 trial similarly found a survival benefit with surgery over watchful waiting in men with localized PC and HR features (8). Likewise, studies of radiotherapy have shown a benefit to radiotherapy plus ADT vs. ADT alone in localized HRPC (9–11). As such, local therapy with surgery or radiation is needed in HRPC but the optimal treatment remains controversial.

### Surgery as primary treatment

Urologists have historically been cautious with surgery for patients with HRPC. While the era of aborted procedures for gross nodal involvement has passed (12), concerns exist over extraprostatic disease leading to treatment failure and need for adjuvant therapies. This coupled with risks of surgery and postoperative functional outcomes have allowed Radiation Oncologists to establish dominance in the primary management of HRPC, with EBRT + ADT or EBRT + BT + ADT as the only recommendations with Category 1 evidence in the NCCN guidelines (3). Despite this, the use of surgery for HRPC is on the rise (13) and owed mostly to retrospective series that show prostate cancer specific survival (PCSS) rates of >90% (14–21). However, a deeper dive into this literature reveals concerning patterns. Namely, the studies are limited by bias inherent to their retrospective design, a prevalent use of adjuvant therapy and heterogeneous outcomes amongst men in the HR group. In a study by Loeb et al., 175 men underwent RP for HRPC with 10-year PCSS of 92%. However, biochemical recurrence-free survival (BCRFS) was 68%, metastasis-free survival (MFS) was 84% and 29% of all patients required some type of hormonal therapy (14). Briganti et al. reviewed 1,366 patients with HRPC who underwent RP at 8 European centers. Only 37% of the patients had specimen-confined disease at final pathology, but 10-year PCSS was significantly higher in this group vs. those with extraprostatic disease (98% vs. 88%, p<0.0001) as was BCRFS (66% vs 47%, p<0.0001). Adjuvant therapy with either ADT or RT was used in 48% of all patients; 66% with extraprostatic disease and 17% with specimen-confined disease (15). A retrospective review by Ward et al. of 5,652 men who underwent RP at a single institution found 842 who had surgery for locally advanced (cT3) disease. PCSS for the entire cohort at 10 years was 90% but 78% of those with pT3 disease received adjuvant therapy, highlighting a risk of local failure and need for multimodal therapy with primary surgery (17). Spahn et al. reported on 550 patients with preoperative HRPC and found an 8-year PCSS of 88%. The importance of stage and surgical margins was revealed as those with pT3a disease or pT3b with negative margins had a PCSS of 92% while those with pT3b disease and positive bladder neck margins had a 5-year PCSS of 60% (20). A review by Djaladat et al. of 358 patients with Gleason 8-10 disease after RP, found significantly better 5-year BCRFS and clinical recurrence free survival in those with Gleason 8 vs. Gleason 9-10 disease (75.5% vs. 71.2%, p=0.01 and 94% vs 86.2%, p=0.02, respectively) (22). Yossepowitch et al explored the divergent outcomes in men considered to have HRPC. They studied 5,960 men who underwent RP to determine how accurately common used definitions of HR disease can predict need for adjuvant therapy, risk of metastases and death from PC. They identified 8 different high-risk subsets with freedom from secondary therapies ranging from 3576% and incidence of death from prostate cancer ranging from 3-11% (18).

Clearly, some patients with HRPC do well after surgery but should it be offered to all men with HRPC? A critical appraisal of the studies above suggests that surgical benefit required adjuvant treatment and was greatest in men with lower or more intermediate risk features. Thus, the answer to the question of ideal treatment options requires a contemporary look at risk groupings.

### Defining “true” high-risk patients

In 2012, Pierorazio et al. sought to identify preoperative characteristics that would predict unfavorable pathology and clinical outcomes after RP. They examined 842 men with Gleason score 8-10 on preoperative biopsy. Unfavorable final pathology (defined as Gleason 8-10 disease and pT3b or N1 disease) was found in 22% of men. Those with unfavorable pathology had worse 10-year BCRFS (4.3% vs. 31%) and half received adjuvant therapy. Despite adjuvant treatment, they still had worse MFS (29.1% vs. 60.9%) and PCSS (52.3% vs 74.7%) when compared to those with favorable pathology. On multivariate logistic regression analysis, a PSA>10ng/mL, cT2b or higher, Gleason 9 or 10, increasing number of cores positive and >50% core involvement were predictive of unfavorable pathology (23). A follow up study from the same institution by Sundi et al proposed a sub-stratification of men with HRPC into two separate groups; HR defined per NCCN guidelines as Gleason 8-10 on biopsy, PSA>20ng/mL or clinical stage ≥ T3 and very high-risk (VHR), defined as primary Gleason pattern 5 on biopsy, ≥ 5 cores with Gleason 8-10 disease or multiple NCCN high-risk features. Of 753 men with NCCN HR disease, 15.1% were found to have VHR disease. These men had significantly worse 10- year MFS (37% vs 78%) and PCSS (62% vs 90%) (24).

These findings prompted an update to the NCCN guidelines in 2015, which now distinguish between HR (≥cT3a or Gleason 8-10 or preoperative PSA>20ng/mL) and VHR PC (cT3B or greater, primary Gleason pattern 5, or 5 or more cores with Gleason 8-10) (3).

The European Urological Association (EAU) discriminates between localized and non- localized HRPC. The first is defined as PSA >20ng/mL or Gleason >7 (International Society of Urological Pathologists, ISUP Grade 4/5) or cT2c, while the second includes cT3a-cT4 with any PSA and any GS (any ISUP score) (4). The America Urological Association defines the HRPC as any patient with PSA≥ 20ng/mL or Gleason score >7 (ISUP Grade 4/5) or cT3, including only the non-localized diseases (Table-1) (5, 6).

The incorporation of VHR criteria into the NCCN guidelines was validated by Pompe et al. who performed a retrospective evaluation of 1,369 VHR cases compared to 2,672 HR ones. Those with VHR disease had higher rates of positive margins (43% vs 32.8%, p<0.001) and positive lymph nodes (40% vs 23.9%, p<0.001) than those with HR disease. Biochemical recurrence occurred within 12 months in 53.7% of those with VHR disease. These patients received adjuvant therapy in 15.2% and salvage in 42% of cases. BCRFS at 8 years was 25.4% in the VHR group and 43.1% in the HR group (p<0.001). Similarly worse differences were seen in 8-year MFS (71.5% vs 86.1%, p<0.001) and PCSS (76% vs 83.7%, p<0.001) in the VHR compared to HR groups, respectively (25).

A recent collaborative study from 3 tertiary centers examined the outcomes after surgery in patients with HR vs VHR disease as defined by the prior report from Sundi et al (26). They included 1,981 men with HRPC and 602 with VHR disease. The rates of positive margins and nodal metastases were significantly greater in the VHR group compared to the HR group (37% vs 25% and 37% vs 15%, respectively, p<0.001). The development of metastases and death from prostate cancer were also significantly higher for those with VHR vs HR disease (HR 2.78, 95% CI 2.083.72 and 6.77 95% CI 2.91-15.7, respectively). Overall, 34 men died from PC and they were more likely to have met VHR vs HR criteria (76% vs 24%, p<0.001).

If the summative conclusion from these studies is that certain men with truly (very) HR disease do worse after surgery, do they also do worse after radiation therapy? Two recent reports give conflicting evidence. Narang et al compared 288 patients with HRPC as defined by the NCCN guidelines and 99 with VHR disease as defined by Sundi et al (27). All men received definitive radiation therapy by a single provider and they found that men in the VHR group did significantly worse. Men with VHR as compared to HR disease had greater biochemical failure (54% vs 35%, p<0.001), distant metastases (34.9% vs 13.4%, p<0.001) and death from PC (18.5% vs 5.9%, p<0.001). However, the median radiation dose received by all patients was 70.2Gy, which is considered sub-standard for men with HR disease given the results of a randomized trial showing the benefits of dose escalated RT (28). Only 75% of VHR and 61% of HR patients received neoadjuvant, concurrent and adjuvant ADT as is recommended by guidelines (3). A more recent report by Saad et al compared 103 patients with VHR and 100 with HR disease per NCCN guidelines. The dose to the prostate was 78-82Gy, to the pelvic lymph nodes was 46-50 Gy and duration of ADT ranged 6 months to 2 years. They found no statistically significant difference in 4- year BCRFS (85% vs 92%), MFS (87% vs 93%) and PCSS (98% vs 100%). However, distant metastases were more common in the VHR group and a PSA≥ 40ng/mL was predictive of biochemical recurrence (HR 3.75) and distant metastases (HR 3.25) (29).

### Surgery vs. radiation

The literature comparing surgery vs radiation in HRPC lacks direct comparison with randomized trials. Several retrospective studies have been performed. While a simple vote count of those favoring surgery or radiation does not provide conclusion, some inferences can be made. In a study of 7,538 men with localized PC in the CAPSURE database, Cooperberg et al found a greater risk of death from PC with EBRT vs RP (HR 3.22, 95% CI 2.16-4.81 vs 2.1 95% CI 1.50 -3.24). This difference was greatest in those men with HRPC (30). While not studying HRPC on its own, Zelefsky et al reported on men with cT1-T3a PC and found that RP had a lower risk of metastases and death from PC as compared to EBRT (31). In a study of men with HRPC, Boorjian et al compared 1,238 men after had RP, 344 with EBRT + ADT and 265 with ADT alone and found similar 10-year PCSS among all groups (92%, 92% and 88%, respectively; p=0.06). There was no difference in risk of metastases or death from prostate cancer but the risk of all causes mortality was greater after EBRT + ADT vs RP (HR 1.60, 95% CI 1.25-2.05) (32). Kishan et al performed a multi-center review of 487 patients with Gleason 9-10 disease on biopsy and compared 230 with EBRT, 87 with EBRT + BT, and 170 with RP. Local salvage was required in 49% of RP patients not receiving adjuvant therapy, 0.9% of EBRT patients and 1.2% of EBRT + BT patients (p <0.0001). The 10-year MFS was higher for EBRT + BT compared to either EBRT or RP (89.8% vs 66.7% vs 61.5%, respectively, p <0.01 for both EBRT + BT vs EBRT and EBRT + BT vs RP) but 10-year PCSS was similar amongst all (80.5% for EBRT, 88.1% for EBRT + BT, and 78.5% for RP, p>0.1). However, a subset analysis of those who received dose escalated radiotherapy showed a significant improvement in PCSS (HR 0.93, 95%CI 0.87-0.99) (33).

The concept of a survival advantage with a dose-escalated boost of radiotherapy was furthered by Kalbasi et al who performed a retrospective analysis of 13,538 men from the National Cancer Database (NCDB). They compared dose-escalated vs standard dose EBRT for men with intermediate or HRPC. Dose escalation was associated with a survival advantage in the HR group (HR 0.82, 95%CI 0.78-0.85) and every 2 Gy increase lead to a 6.3% reduction in the risk of death (34). Moreover, results of the ASCENDE-RT trial have strengthened evidence for the role of BT in men with HRPC. In this study, all men with HRPC received 12 months of ADT and EBRT with 46Gy followed by either a dose-escalated boost to 78Gy or a low-dose--rate brachytherapy boost. They found that men randomized to BT experienced a 2-fold decrease in risk of biochemical recurrence as compared to those who received a boost with EBRT (HR 2.04, p=0.004) (35). There have since been conflicting reports from the NCDB on the role of EBRT + BT boost as compared to RP (36, 37), but they are limited by the inherent lack of granularity in the database where disease specific survival and progression are not available and true rates of ADT use and radiation dosage are unclear. In a recent report by Tilki et al, 639 men with Gleason 9-10 disease who had either RP (n=559) or EBRT + ADT + BT (n=80) were compared. There was no difference in risk of death from prostate cancer amongst the two groups (HR 1.33, 95%CI 0.49-3.64), but 15.7% of RP patients received adjuvant EBRT, 8.8% received ADT and 8.9% received both. This suggests that an equivalent survival in primary surgery vs primary radiotherapy comes from the adjuvant treatment surgical patients receive (38). Concerns over increased side effects with BT are real (39), but the radiotherapy literature has put forth new efforts to decrease morbidity while still providing what appears to be an effective treatment boost. An ongoing trial investigating the role of focal dose escalation to MRI targeted lesions has shown no increase in toxicity when compared to standard radiation doses (40).

## CONCLUSIONS

Several guideline-approved options exist in the management of HRPC. While data indicate that local treatment is needed, the appropriate role of surgery vs radiation remains less clear. In consideration of efficacy, RP certainly seems to be efficacious in a subset of patients with HRPC. However, retrospective series suggests the benefit is greatest in patients with lower risk disease. In the VHR group alone, RP is plagued by local failure and radiotherapy by distant failure. For this reason, trials are underway to investigate the added benefit of systemic therapy with radiotherapy in localized disease (41) and greater efforts at improving local control with surgery are needed. Side effects need to be considered as well. While radiotherapy comes with greater irritative voiding symptoms, surgery is associated with decrease in sexual function and urinary continence when compared to EBRT (42). Again, the impetus is on Urologists to improve these outcomes without sacrificing local control of disease. As it stands, the surgical management of HRPC is a multimodal one where patients should expect the use of adjuvant therapies such as radiation or ADT. The conflicting results of several retrospective series merit further investigation with clinical trials (43).
